# Variations in the Temperature-Humidity Index and Dorsal Fat Thickness during the Last Trimester of Gestation and Early Postpartum Period Affect Fertility of* Bos indicus* Cows in the Tropics

**DOI:** 10.1155/2018/2360430

**Published:** 2018-07-24

**Authors:** Ramiro F. Díaz, Carlos S. Galina, Sandra Estrada, Felipe Montiel, Gloriana Castillo, Juan José Romero-Zúñiga

**Affiliations:** ^1^Escuela de Medicina Veterinaria, Universidad San Francisco de Quito, Diego de Robles S/n, Cumbayá, Ecuador; ^2^Departamento de Reproducción, Facultad de Medicina Veterinaria y Zootecnia, Universidad Nacional Autónoma de México, Ciudad Universitaria, 04510 México DF, Mexico; ^3^Programa de Investigación en Medicina Poblacional, Escuela de Medicina Veterinaria, Universidad Nacional de Costa Rica, Código Postal 304-3000, Heredia, Costa Rica; ^4^Departamento de Reproducción, Facultad de Medicina Veterinaria, Universidad Veracruzana, Circunvalación S/n Esquina Yáñez, Código Postal 91710, Veracruz, Mexico

## Abstract

In order to measure the influence of the temperature-humidity index (THI) and the variation of fat thickness on reproductive performance, ninety-two* Bos indicus* cows kept under grazing conditions were used in two farms in Veracruz (Mexico) and Puntarenas (Costa Rica). THI was calculated with the average environmental temperature and relative humidity. Measurements of fat thickness (FAT) were taken two weeks apart from the last trimester of gestation to approximately 80 days postpartum (dpp). Natural breeding was used in both farms. Time to conception was calculated based on the interval from days at risk to conception (DRC), which had to be at least 28 dpp. THI was between 65.4 ± 2.9 and 73.2 ± 1.5 in Veracruz and 75.4 ± 0.26 and 76.5 ± 0.55 in Puntarenas. Variations in THI were observed in Puntarenas whereas in Veracruz THI variations were more prominent. In Veracruz, loss of fat during the last trimester of gestation was on average 8.5%, whereas in the postpartum period it was 18.4% (P = 0.042). In Puntarenas, the variation in the last trimester of gestation was on average 18.7% and in the postpartum period was 10.5% (P = 0.012). The relative change in FAT in Veracruz was 36.7%, and in Puntarenas it was 29.3%. Overall, 60% of the cows became pregnant. FAT decreased the interval of DRC (R^2^=0.06; P=0.033) with a high relationship (R^2^=0.76; P< 0.0001) between THI and time to conception, in both farms. In conclusion, THI levels influence the reproductive performance in early postpartum period affecting DRC.

## 1. Introduction

The effect of the environment on productive and reproductive activities has been the subject of much study [1–5]. Stress in cattle due to the harsh environmental conditions has several effects on reproductive physiology, such as the presence of overt signs of estrus, rate of ovulation, and embryo survival [2, 6]. Villa-Mancera et al. [7], reported a direct effect of the temperature-humidity index (THI) on conception rate, registering lower levels of fertility during the summer months compared to the winter. It is well-known that* Bos indicus *cattle raised under tropical conditions show prolonged calving intervals [8, 9], which are attributed to long periods of postpartum anestrus [10]. Fat thickness (FAT) has been shown to be a reliable indicator of the level of energy reserves in the animal [11]. There is evidence suggesting that the FAT during the last trimester of pregnancy is associated with reproductive capacity [12–14]. Therefore, early resumption of ovarian activity is closely related to the ability of the dam to recover from the negative energy balance caused by stress during calving [8] and the inability to cope with extreme environmental conditions [15]. Thus, the objective of this study was to measure the effect of THI and fat thickness during the last trimester of pregnancy and early postpartum period on the time to conception of* Bos indicus* cows bred on natural mating in the dry tropics of Mexico and Costa Rica.

## 2. Materials and Methods

The Animal Care Internal Committee (CICUAE) of the Faculty of Veterinary Medicine and Zootechnics of the National Autonomous University of Mexico approved the methods used during the present work in accordance with the Code of Ethics of the World Medical Association (Declaration of Helsinki).

### 2.1. Location

This study was carried out simultaneously in two farms, located at 19°10′36.93′′ N and 96°16′3.20′′ W in the municipality of Manlio Fabio Altamirano of the state of Veracruz, Mexico, with a precipitation and temperature average of 108.4 mm and 27.05°C, respectively. The other farm is at 10°2′39.04′′ N and 84°54′23.44′′ W, situated in the province of Puntarenas, Costa Rica, with a precipitation and temperature average of 111.87 mm and 27.42°C, respectively. The study was carried out from February to August 2015. Both farms are located in the zone considered as dry tropics (National Institute of Statistic and Geography, http://www.inegi.org.mx; National Meteorological Institute of Costa Rica, http://www.imn.ac.cr.) The THI for each farm was calculated monthly considering the average environmental temperature and relative humidity of the period in which the study was conducted, applying the equation proposed by García-Ispierto et al. [16]:

(1) $$\begin{array}{c} THI=\left(\;0.8\times T+\left(\;\frac{RH}{100}\;\right)\times \left(\;T-14.4\;\right)+46.4\;\right) \end{array}$$

where THI is the temperature-humidity index; *T* is the mean temperature expressed in °C; and *RH* is the average relative humidity expressed in percentage. Unfortunately, wind speed and solar radiation were not considered; it was assumed that THI was the best indicator of animal heat stress [12–14]. As it was an observational study in private farms, data on climatic factors were taken from weather stations nearby not further than 10 km. THI levels over 74 were considered indicators of heat stress.

In addition, the photoperiod (number of daylight hours) in each farm during the study was measured. This measure is known as* maximum duration of insolation* and is estimated as follows [15]:

(2) $$\begin{array}{c} N=\left(\;\frac{24}{\pi }\;\right)\omega_{s} \end{array}$$

where* N* is the maximum duration of insolation; and *ω*_s_ is the angle of solar radiation at sunset time, calculated with the following equation:

(3) $$\begin{array}{c} \omega_{s}=\left(\;\frac{\pi }{2}\;\right)-\arccos\left[\;-\tan\left(\;\varphi \;\right)\tan\left(\;\delta \;\right)\;\right] \end{array}$$

where *φ* is the latitude expressed in radians; and *δ* is the solar declination expressed in radians, calculated as

(4) $$\begin{array}{c} \delta =0.409\times \mathrm{sen}\left\{\;\left[\;\left(\;\frac{2\pi }{365}\;\right)J\;\right]-1.39\;\right\} \end{array}$$

where J is the number of days in the year between 1 (1 January) and 365 (31 December).

### 2.2. Animals

Ninety-two multiparous Brahman cows in their last trimester of gestation were used. Animals were managed under an extensive grazing system. In Veracruz (n= 37), paddocks contained pastures such as Pangola* (Digitaria decumbens), *Guinea* (Panicum maximum), *Estrella* (Cynodon plectostachyus), *and Gramilla* (Cynodon dactylon)*. In Puntarenas (n= 55), pastures were mainly Braquiaria (*Brachiaria brizantha) *and* Guinea (Panicum maximum).* The animals were not given supplementary feed during this period, although they had access* ad libitum* to water and mineral salts. The body condition of the animals at the start of the experiment was on average 3.5 in Veracruz and 3.8 in Costa Rica using the scale of 1–9 (1 = emaciated, 9= obese).

### 2.3. Fat Thickness

In both farms, all measurements of fat thickness (FAT), expressed in centimeters (cm), were taken two weeks apart from the beginning of the last trimester of gestation to around 80 days postpartum (dpp), using the ALOKA ProSound 2 ultrasound equipment with a convex array transducer 3.5 MHz. The transducer was placed horizontally in the caudal region between the iliac and the ischial tuberosity [11].

### 2.4. Evaluation of Time to Conception

Natural breeding was used in both farms; the moment when the bull joined the herd was an important factor for evaluation of reproductive performance. The calving season in both farms was between April and June. On each farm, bull andrological examinations were performed and their fertility was tested. Time to conception was calculated based on the interval from days at risk to conception (DRC), corresponding to the number of days that a cow has higher probability to get pregnant following first exposure to the bull. The DRC starting 28 dpp to the day when the bull joined the herd was established and was calculated based on the interval from partum to conception (PCI) centered on serial ultrasounds to measure the size of the embryo using the scale proposed by Rosiles et al. [17]. Cows were divided into three subgroups in relation to the time when the bull was introduced to the herds:

Calved when the bull joined the herd ≥ 28 dpp (Veracruz n=9; Puntarenas n= 47)

Calved when the bull joined the herd < 28 dpp (Veracruz n= 17; Puntarenas n=8)

Still pregnant when the bull joined the herd of cows (Veracruz; n= 11).

Using this arbitrary division, for animals in subgroups 1 and 2, DRC was calculated by subtracting PCI from calving to the day the bull joined the herd of cows by applying the following formula:

(5) $$\begin{array}{c} \begin{array}{c} DRC=PCI-\text{calving days until the herd was exposed to the bull} \end{array} \end{array}$$

For the cows that were already pregnant when the bull joined the herd, DRC was calculated subtracting 28 from the PCI:

(6) $$\begin{array}{c} DRC=PCI-28 \end{array}$$

The bull was with the herds for 65 days. Pregnancy diagnosis was performed in those cows with more than 28 days postpartum (dpp) using an ultrasound ALOKA ProSound 2 equipped with a linear transducer of 7.5 MHz. In addition, the percentage of pregnant cows was measured depending on the length of exposure to the bull.

### 2.5. Statistical Analysis

The interval of days at risk to conception for all groups of cows for each farm was calculated using the arithmetic mean and standard deviation. The percentage of cows that were pregnant on different days after the entry of the bull was compared using a Student T-test for each point of measuring. Changes in back fat thickness (cm) during the last trimester of pregnancy and until the moment of gestation, or the time when observations finished, were analyzed by descriptive statistics and a Student T-test for each point of measuring. In all cases, in order to establish possible differences, the 95% confidence interval was also calculated. Conversely, total relative change in back fat (ΔFAT= [initial measure - final measure]/initial measure) was determined. This variable was considered as either continuous or categorical and divided into high variation (≥ 66th percentile), medium (between 33th and 66th percentiles), and low (≤ 33th percentile). Percentiles were established specifically for each farm.

The relationship between THI and daylight hours with the relative changes of FAT and DRC were established by simple lineal regression and one-way ANOVA to compare the means of FAT relative change, categorized with respect to DRC.

Then, again, the percentage of pregnant animals was measured depending on the time of exposure to the bull. Comparisons of percentages by means of chi-square test and 95% confidence interval were performed for each group of cows. Thus, an analysis was carried out in those animals pregnant within the first 28 days of exposure to the bull, those that became pregnant within 56 days after the entry of the male, and finally those animals that became pregnant within 65 days following the entrance of the bull.

Finally, a nonparametric survival analysis was performed using Kaplan-Meier curves to evaluate the effect of total relative FAT change on the curves of DRC survival analysis, using the log rank as a statistical test for the curves comparison and total relative FAT change in a categorical form (high, medium, and low change).

In all statistical processes, a significance value of 5% was used as the point of statistical decision. All the analyses were performed using IBM SPSS 22 and Infostat 2016.

## 3. Results

### 3.1. Temperature-Humidity Index and Daylight Hours

**Figure 1:**
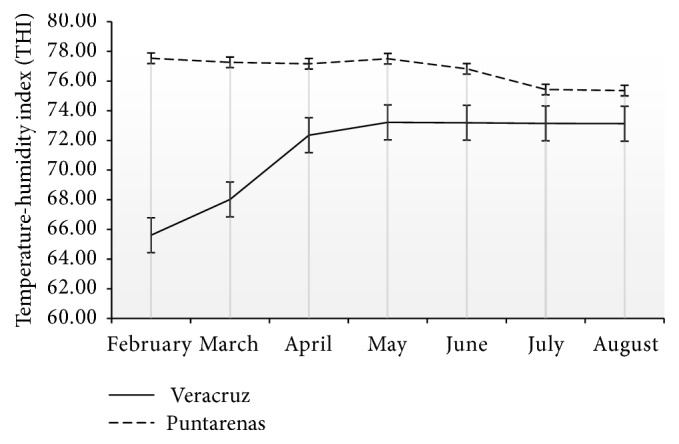
Temperature-humidity index (THI) for Veracruz and Puntarenas from the months of February to August 2015.

In Veracruz, THI levels were between 65.4 ± 2.9 and 73.2 ± 1.5 and in Puntarenas between 75.4 ± 0.26 and 76.5 ± 0.55 (Figure 1).

**Figure 2:**
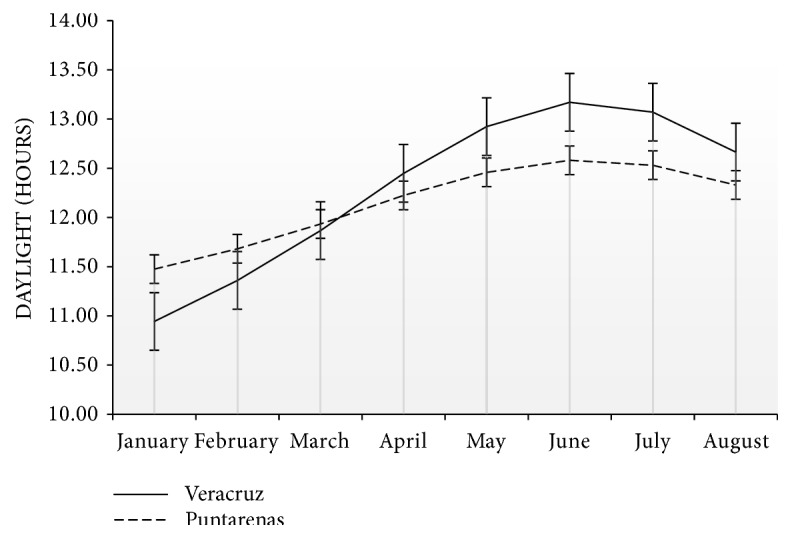
Daylight/hours in Veracruz and Puntarenas from January to August 2015. Fat thickness (FAT).

Considering their different geographic locations, the farms had diverse daylight changes during the study. In January, Veracruz had fewer than 11 daylight hours and more than 13 hours in June, while Puntarenas had more than 11 hours in January and by June the daylight hours did not reach 13 (Figure 2).

**Figure 3:**
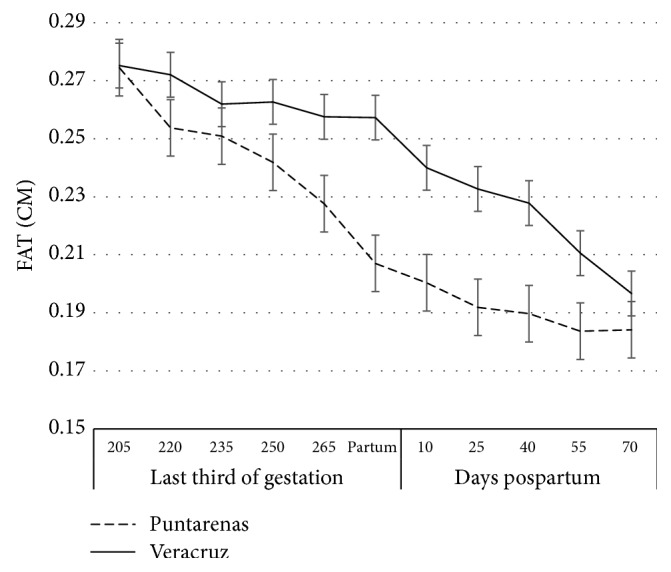
Back fat thickness in all cows, specifically in Veracruz and Puntarenas cows (error bars are presented).

In both farms, FAT decreased gradually during all the study. On average, cows initiated with 0.28 cm, reducing to 0.23 cm at calving and down to 0.20 cm at the end of the observation period. General and specific changes per farm are shown in Figure 3.

Body fat changes showed differences between farms. In Veracruz, loss of FAT during the last trimester of gestation was on average 8.5% and 18.4% in the postpartum period (P = 0.042). In Puntarenas, the variation in the last trimester of gestation was on average 18.7% and in the postpartum period 10.5% (P = 0.012). In total, the relative change in BF thickness was greater in Veracruz, 36.7%, than in Puntarenas, 29.3% (Figure 3).

### 3.2. Time to Conception

In Veracruz, the bull joined the herd when 30% (n= 11) of the cows were still pregnant; 46% (n=17) were less than 28 dpp and 24% (n= 9) were more than 28. In Puntarenas, the bull joined the herd when all cows had calved; 27% (n= 15) were between 15 and 30 dpp; 29% (n= 16) were between 31 and 52 dpp; 24% (n= 13) were between 53 and 60 dpp; and 20% (n= 11) were between 61 and 72 dpp. In total, 60% conceived during the observation period, in Veracruz, as well as 49% and 67% in Puntarenas. In Veracruz, none of the nonpregnant animals conceived during the first 28 days after the bull was with the herd. In contrast, at 56 days after bull exposure, 47% had conceived (P<0.05). The percentage of pregnancies in Puntarenas for the first 28 days after bull exposure was 47%. Furthermore, by 56 days 48% had become pregnant. No pregnancies were observed after this period.

When grouping the animals on the farms according to the time postpartum when they are at risk being pregnant, conception rates were 39% for the first 28 days, 28% for the period from 29 to 56 days, and 31% for the cows becoming pregnant after this date. Global average of PCI was 78.5 ± 19.0 dpp. Specifically, the average of PCI per farm was 91.6 ± 14.8 and 72.2 ± 17.6 for Veracruz and Puntarenas.

### 3.3. Relationship between Temperature-Humidity Index, Changes in Fat Thickness, and Time to Conception

The loss of FAT decreased the interval of DRC (R^2^ =0.06; P=0.033). This same tendency was observed within farms, although without statistical significance (P= 0.7931 and P=0.4207 for Puntarenas and Veracruz, respectively). There was a strong relationship (R^2^ = 0.76; P< 0.0001) between THI during postpartum period and time to conception.

**Figure 4:**
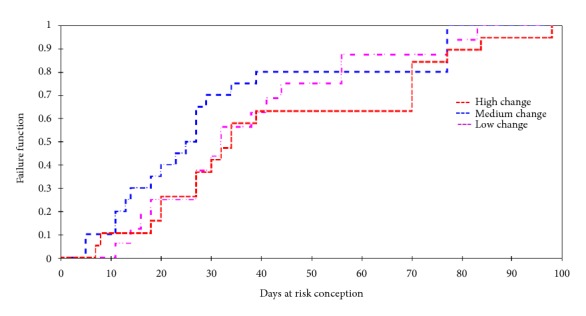
Kaplan-Meier's curve for time to conception in* Bos indicus* cows from two farms located in the dry tropical climate of Mexico and Costa Rica. No major differences were observed in the medium and low FAT changes whereas the cows with high changes were statistically different (P<0.05).

Significant differences were not found (P > 0.05) between the averages of DRC analyzed by linear regression comparing high, medium, and low FAT levels. This was confirmed by the Kaplan-Meier test, where cows with greater FAT loss had a more prolonged survival function for PCI, whereas the cows that showed medium and low FAT loss became pregnant faster (Chi2 log rank test = 1.786, P =0.41). The mean survival (DRC) was 34 days for cows with high FAT, while for medium and low FAT it was 26 and 32 days, respectively (P = 0.17; Figure 4).

## 4. Discussion

It is important to remark that the comparison between farms is just to illustrate the differences of than factors studied; thus, animals from both farms lost back fat thickness (FAT) during the last trimester of gestation, as well as in the postpartum period. The change in their metabolic status may be associated with specific events taking place towards the end of gestation and the beginning of lactation. Bovines, as do other mammals, exhibit maximum fetal growth in the last trimester of gestation [18, 19]. Consequently, the nutritional requirements of the fetus are greatest at this time [18]. On the other hand, during the early postpartum period, the animals suffer an imbalance in energy status, due to the beginning of the lactation period, coupled with intensive suckling. This causes the dam to mobilize body energy reserves, which triggers negative energy balance [20]. This was confirmed in the present study, since the animals from the farm in Veracruz had greater back fat thickness loss during the postpartum period as opposed to the prepartum period (P = 0.042). In earlier research, a poor correlation has been found between body condition and fat thickness, and several authors have confirmed that dorsal fat thickness is an indicator of nutritional status far more reliable than body condition [11, 21].

The cows in the farm at Puntarenas lost less fat in the postpartum period, compared to prepartum period (19.2% versus 11.3%, respectively; P = 0.012). This change in the variation of fat loss may be associated with differences in daylight duration. Veracruz had greater daylight hour variation than Puntarenas during the experiment. Light increases water-soluble carbohydrate levels and decreases the concentration of lignin in the tropical pastures causing an increase in digestibility [22]. The last trimester of gestation in Veracruz occurred in the majority of animals, during the period of shorter daylight hours, suggesting that the digestibility of the pastures could not have been optimal during this period. Unfortunately, no measurements of grass quality were undertaken; however, there is ample evidence [22–28] demonstrating the sensitivity of pastures to the number of light hours. On the contrary, the time of year with more daylight hours coincided in most of the animals with the postpartum period. Thus, an increase in hours of light would likely have improved pasture digestibility.

Then again, for the last four decades, the temperature-humidity index has become a standard in cattle management practices [29–31] to assess livestock comfort. According to climatological data for the year in which this study was conducted and based on the information published by Arias et al. [32] in Veracruz they were not subjected to heat stress (state of comfort < 74 THI) but only during last trimester of gestation. Once calving started, THI increased towards the alert area on the THI scale (between 74 and 78 THI) [32], probably causing stress in the animals and affecting their feed intake. Instead, animals in Puntarenas were constantly in the alert zone and without drastic changes in THI, suggesting a state of physiological adaptation to the stable environmental conditions prevailing during most of the year. The present investigation was carried out in animals at pasture, which makes it difficult to measure items such as respiratory rate and rectal temperature.

Conception rates were 49% in Veracruz and 67% in Puntarenas giving a mean of 60%, in agreement with the 41 to 70% for* Bos indicus* cows previously reported [33–35]. The PCI average of both farms was 78.5 ± 19.0 days, similar to that observed by Schramm et al. [36], who reported a calving-conception interval in* Bos indicus* of 81 ± 9 and 79 ± 8 days among their experimental groups. However, PCI was different for the two farms, longer in Veracruz than in Puntarenas (91.6 and 72.2, respectively). Likewise, DRC was greater in Veracruz than in Puntarenas (68.9 and 22.5, respectively). The postpartum time when the animals entered the reproductive program may have a direct effect on fertility, whereas, in Veracruz, 75% of cows had less than 28 dpp or were pregnant when the bull joined the herd; 100% of the cows in Puntarenas had already calved. Consequently, 85% of the animals in Puntarenas were already at risk of conception in the first 28 days after the bull joined the herd, in comparison to only 24% in Veracruz. Previous reports indicate that the time required for cows to become pregnant relates to the average number of days postpartum when the bull joins the herd [37, 38].

THI may have a direct effect on fertility [7, 16] explaining in part the differences in time to conception between the two farms. In Veracruz, there were drastic changes in THI during the study, from 65.4 in the months of February and March to an average of 73.2 in late June, July, and August with conditions varying from comfort to alert [32]. In Puntarenas, in contrast, THI remained stable with an average of 76 for the duration of the study. One shortcoming of the present study is the variation in the numbers of animals at risk of becoming pregnant when the bull joined the herd. However, even with this limitation, results suggest it is important for future studies to not only specify the exact location but also provide precise information about daylight hours and THI.

Animals with greater FAT variation tended to delay pregnancy longer compared to those with medium or low variation. Samadi et al. [39] reported that animals consuming better quality pastures between the 6th and 7th month of gestation resumed ovulation earlier. Therefore, it is reasonable to assume that animals with superior energy reserves during the last trimester of gestation and in early postpartum are likely to conceive in a shorter time after calving than animals mobilizing their fat reserves to achieve this goal. Insufficient intake of calories during the last stages of gestation affects fertility even when energy intake is adequate during lactation [12, 40–42]. Hess et al. [43], in an extensive review, reported that the level of feed intake during prepartum period has a greater effect on postpartum anestrus than does feed intake after calving. This indicates that FAT variation during the last trimester of gestation directly influences postpartum reproductive development.

On the other hand, THI has been shown to relate strongly with fertility, particularly during the postpartum period (R^2^ = 0.76; P > 0.0001). These results are similar to those previously reporting a close relationship of climatic factors with conception rate, progesterone levels, duration, and intensity of estrus [7, 16, 44, 45]. The change to alert levels of THI in Veracruz during the summer months (between 74 and 78) could help to explain the lower fertility encountered. These results also agree with studies conducted in European cattle; for instance, Petersson et al. [45] found that the interval between parturition and first luteal activity is greater when calving takes place during winter and the return to the reproductive program occurs in the summer and optimum fertility. García-Ispierto et al. [16] observed that as THI increases around 3 days after artificial insemination, conception rate decreases. Some reports also indicated an effect on conception rate [46] and days open [15] when fluctuations in THI occur in the locality. In Veracruz, increasing levels of THI of more than 7 points occurred when 70% of cows were at risk of pregnancy. However, in Puntarenas, THI showed only a 1.1 variation during the study suggesting that animals probably did not have to adapt to drastic changes in this important parameter [29, 47].

Finally, it is worth pointing out the differences in the reproductive status of cows when the bull enters the breeding program. It is common management practice to employ animals regardless of whether they are likely to conceive or not which was the case of the farm in Veracruz. This policy needs revision.

## 5. Conclusions

Temperature-humidity index levels in tropical areas could influence the reproductive development of animals in early postpartum affecting conception rate. The loss of fat in either the prepartum or postpartum periods affects time to conception being shorter in those animals losing less FAT during the same periods. However, further research is in demand before any conclusions can be safely drawn.

## Data Availability

Most of the analyses that were carried out in this investigation were made with the data that can be found in the link “https://drive.google.com/file/d/1NFDCmIppF20hz4IDe__v_e4d8FZX8fbM/view?usp=sharing” or provided by the corresponding author upon request.

## References

[1] Azzam S. M., Kinder J. E., Nielsen M. K. (1993). Environmental effects on neonatal mortality of beef calves. *Journal of Animal Science*.

[2] Silva C. F., Sartorelli E. S., Castilho A. C. S. (2013). Effects of heat stress on development, quality and survival of Bos indicus and Bos taurus embryos produced in vitro. *Theriogenology*.

[3] Amundson J. L., Mader T. L., Rasby R. J., Hu Q. S. (2006). Environmental effects on pregnancy rate in beef cattle. *Journal of Animal Science*.

[4] Schüller L. K., Burfeind O., Heuwieser W. (2014). Impact of heat stress on conception rate of dairy cows in the moderate climate considering different temperature-humidity index thresholds, periods relative to breeding, and heat load indices. *Theriogenology*.

[5] Meza-Herrera C. A., Vicente-Pérez A., Osorio-Marín Y. (2015). Heat stress, divergent nutrition level, and late pregnancy in hair sheep: effects upon cotyledon development and litter weight at birth. *Tropical Animal Health and Production*.

[6] Hansen P. J. (2007). Exploitation of genetic and physiological determinants of embryonic resistance to elevated temperature to improve embryonic survival in dairy cattle during heat stress. *Theriogenology*.

[7] Villa-Mancera A., Méndez-Mendoza M., Huerta-Crispín R., Vázquez-Flores F., Córdova-Izquierdo A. (2011). Effect of climate factors on conception rate of lactating dairy cows in Mexico. *Tropical Animal Health and Production*.

[8] Montiel F., Ahuja C. (2005). Body condition and suckling as factors influencing the duration of postpartum anestrus in cattle: A review. *Animal Reproduction Science*.

[9] Escrivão R. J. A., Webb E. C., Garcês A. P. J. T. (2009). Effects of 12 hour calf withdrawal on conception rate and calf performance of Bos indicus cattle under extensive conditions. *Tropical Animal Health and Production*.

[10] Baruselli P. S., Reis E. L., Marques M. O., Nasser L. F., Bó G. A. (2004). The use of hormonal treatments to improve reproductive performance of anestrous beef cattle in tropical climates. *Animal Reproduction Science*.

[11] Schröder U. J., Staufenbiel R. (2006). Methods to determine body fat reserves in the dairy cow with special regard to ultrasonographic measurement of backfat thickness. *Journal of Dairy Science*.

[12] Wettemann R. P., Lents C. A., Ciccioli N. H., White F. J., Rubio I. (2003). Nutritional- and suckling-mediated anovulation in beef cows. *Journal of Animal Science*.

[13] Galindo J., Galina C. S., Estrada S., Romero J. J. (2013). Effect of changes in body weight, body condition and back fat during last month of pregnancy on the reproductive efficiency of *Bos indicus* cows in the tropics of Costa Rica. *Open Journal of Veterinary Medicine*.

[14] Guzmán A., Gonzalez-Padilla E., Garcés-Yepez P. (2016). Increased body condition score through increased lean muscle, but not fat deposition, is associated with reduced reproductive response to oestrus induction in beef cows. *Animal*.

[15] Boni R., Perrone L. L., Cecchini S. (2014). Heat stress affects reproductive performance of high producing dairy cows bred in an area of southern apennines. *Livestock Science*.

[16] García-Ispierto I., López-Gatius F., Bech-Sabat G. (2007). Climate factors affecting conception rate of high producing dairy cows in northeastern Spain. *Theriogenology*.

[17] Rosiles V. A., Galina C. S., Maquivar M., Molina R., Estrada S. (2005). Ultrasonographic screening of embryo development in cattle (Bos indicus) between days 20 and 40 of pregnancy. *Animal Reproduction Science*.

[18] Bell A. W. (1995). Regulation of organic nutrient metabolism during transition from late pregnancy to early lactation. *Journal of Animal Science*.

[19] Guedon L., Saumande J., Desbals B. (1999). Relationships between calf birth weight, prepartum concentrations of plasma energy metabolites and resumption of ovulation postpartum in limousine suckled beef cows. *Theriogenology*.

[20] Grummer R. R., Mashek D. G., Hayirli A. (2004). Dry matter intake and energy balance in the transition period. *Veterinary Clinics of North America: Food Animal Practice*.

[21] Galindo J., Galina C. S., Estrada S., Romero J. J., Alarcón M., Maquivar M. (2013). Effect of changes in body weight, body condition and back fat during last month of pregnancy on the reproductive efficiency of *Bos indicus* cows in the tropics of Costa Rica. *Open Journal of Veterinary Medicine*.

[22] Van Soest P. J., Mertens D. R., Deinum B. (1978). Preharvest factors influencing quality of conserved forage. *Journal of Animal Science*.

[23] Carlson G. E. (1965). *Photoperiodic control of adventitious stem initiation on roots. Crop Sci*.

[24] Heinrichs D. H., Nielsen K. F. (1966). Growth response of alfalfa varieties of diverse genetic origin to different root zone temperatures. *Canadian Journal of Plant Science*.

[25] Robertson G. W. (1966). The light composition of solar and sky spectra available to plants. *Ecology*.

[26] Sato K. (1971). Growth and Development of Alfalfa Plant under Controlled Environment: I. The effects of daylength and temperature on the growth and chemical composition. *Proceedings of the Crop Science Society of Japan*.

[27] Sato K. (1974). Growth and Development of Alfalfa Plant under Controlled Environment: III. The effects of photoperiod and temperature on the growth and anatomical features of photosynthetic tissues. *Proceedings of the Crop Science Society of Japan*.

[28] McCarthy B., Delaby L., Pierce K. M. (2016). The multi-year cumulative effects of alternative stocking rate and grazing management practices on pasture productivity and utilization efficiency. *Journal of Dairy Science*.

[29] Berman A., Horovitz T., Kaim M., Gacitua H. (2016). A comparison of THI indices leads to a sensible heat-based heat stress index for shaded cattle that aligns temperature and humidity stress. *International Journal of Biometerology*.

[30] Khalifa H. H. (2003). Bioclimatology and adaptation of farm animals in a changing climate. *Interactions between Climate and Animal Production. Proceedings of the Symposium*.

[31] Gaughan J. B., Mader T. L., Holt S. M., Lisle A. (2008). A new heat load index for feedlot cattle. *Journal of Animal Science*.

[32] Arias R. A., Mader T. L. Y., Escobar P. C. (2008). Factores climáticos que afectan el desempeño productivo del ganado bovino de carne y leche. *Archivos de Medicina Veterinaria*.

[33] Rekwot P. I., Oyedipe E. O., Mukasa-Mugerwa E., Sekoni V. O., Akinpelumi O. P., Anyam A. A. (1999). Fertility in zebu cattle (Bos indicus) after prostaglandin administration and artificial insemination. *The Veterinary Journal*.

[34] Rekwot P. I., Ogwu D., Sekoni V. O., Oyedipe E. O. (2000). Serum progesterone profiles of zebu cattle (*Bos indicus*) in relationship to conception and repeat breeding after artificial insemination. *Animal Reproduction Science*.

[35] Sales J. N. S., Carvalho J. B. P., Crepaldi G. A. (2015). Effect of circulating progesterone concentration during synchronization for fixed-time artificial insemination on ovulation and fertility in *Bos indicus* (Nelore) beef cows. *Theriogenology*.

[36] Schramm R. D., Roberge S., Reeves J. J. (1991). Enclomiphene does not alter the postpartum interval of suckled beef cows. *Journal of Animal Science*.

[37] Molina R., Galina C. S., Maquivar M., Estrada S., Chávez A., Díaz G. S. (2003). Pregnancy rate in Zebu cows with two different postpartum intervals exposed to a two-bull rotational system. *Veterinary Research Communications*.

[38] Berardinelli J. G., Joshi P. S. (2005). Introduction of bulls at different days postpartum on resumption of ovarian cycling activity in primiparous beef cows. *Journal of Animal Science*.

[39] Samadi F., Phillips N. J., Blache D., Martin G. B., D'Occhio M. J. (2013). Interrelationships of nutrition, metabolic hormones and resumption of ovulation in multiparous suckled beef cows on subtropical pastures. *Animal Reproduction Science*.

[40] Randel R. D. (1990). Nutrition and postpartum rebreeding in cattle. *Journal of Animal Science*.

[41] Short R. E., Bellows R. A., Staigmiller R. B., Berardinelli J. G., Custer E. E. (1990). Physiological mechanisms controlling anestrus and infertility in postpartum beef cattle. *Journal of Animal Science*.

[42] Dunn T. G., Moss G. E. (1992). Effects of nutrient deficiencies and excesses on reproductive efficiency of livestock.. *Journal of Animal Science*.

[43] Hess B. W., Lake S. L., Scholljegerdes E. J. (2005). Nutritional controls of beef cow reproduction. *Journal of Animal Science*.

[44] Gwazdauskas F. C., Thatcher W. W., Kiddy C. A., Paape M. J., Wilcox C. J. (1981). Hormonal patterns during heat stress following PGF2*α*-tham salt induced luteal regression in heifers. *Theriogenology*.

[45] Petersson K.-J., Strandberg E., Gustafsson H., Berglund B. (2006). Environmental effects on progesterone profile measures of dairy cow fertility. *Animal Reproduction Science*.

[46] Mellado M., Sepulveda A., Meza-Herrera C. (2013). Effects of heat stress on reproductive efficiency in high yielding Holstein cows in a hot arid environment. *Revista Colombiana de Ciencias Pecuarias*.

[47] Kumar A., Waiz S. A., Sridhar Goud T. (2016). Assessment of adaptability of zebu cattle (*Bos indicus*) breeds in two different climatic conditions: using cytogenetic techniques on genome integrity. *International Journal of Biometerology*.

